# Telomere Length and Biological Aging: The Role of Strength Training in 4814 US Men and Women

**DOI:** 10.3390/biology13110883

**Published:** 2024-10-30

**Authors:** Larry A. Tucker, Carson J. Bates

**Affiliations:** College of Life Sciences, Brigham Young University, Provo, UT 84602, USA

**Keywords:** biological aging, therapeutic exercise, cell senescence, physical activity, oxidative stress

## Abstract

Telomere length is an index of cellular aging. Healthy lifestyles are associated with reduced oxidative stress and longer telomeres, whereas unhealthy behaviors are related to shorter telomeres and greater biological aging. This investigation was designed to determine if strength training accounted for differences in telomere length in a random sample of 4814 US adults. Data from the National Health and Nutrition Examination Survey (NHANES) were employed to answer the research questions using a cross-sectional design. Time spent strength training was calculated by multiplying days of strength training per week by minutes per session. Participation in other forms of physical activity was also calculated based on reported involvement in 47 other activities. Weighted multiple regression and partial correlation were used to calculate the mean differences in telomere length across levels of strength training, adjusting for differences in potential confounders. With the demographic covariates controlled, strength training and telomere length were linearly related (F = 14.7, *p* = 0.0006). Likewise, after adjusting for all the covariates, the linear association remained strong and significant (F = 14.7, *p* = 0.0006). In this national sample, 90 min per week of strength training was associated with 3.9 years less biological aging, on average. Regular strength training was strongly related to longer telomeres and less biological aging in 4814 US adults.

## Simple Summary

Telomeres cap the ends of chromosomes. The length of telomeres is highly related to chronological age. As people age, their telomeres become shorter. Shorter telomeres put people at a greater risk of premature disease and death. A healthy lifestyle tends to preserve telomeres, whereas unhealthy practices cause increased biological aging and shorter telomeres. This study investigated the extent to which regular strength training is related to the length of telomeres in 4814 US men and women who were representative of the US adult population. Participants gave blood, and the length of telomeres in their blood cells was precisely measured. Participants also reported how often they engaged in exercises to strengthen their muscles. The findings showed that adults who strength trained regularly had significantly longer telomeres and therefore less biological aging than adults who did not strength train, even after taking into account many factors, including their age, sex, race, income, household size, smoking, body size, and participation in physical activities other than strength training.

## 1. Introduction

Strength training used to be a muscle-building activity for brawny men who competed in athletics, such as football, wrestling, and powerlifting. These men needed significant levels of strength and muscularity for an advantage in their sport, and their mesomorphic structures responded favorably to heavy resistance training with barbells and dumbbells [1]. 

In addition to competitive athletes, research shows that strength training has significant health benefits for the average individual. Studies have demonstrated that relatively brief sessions of regular resistance training can increase strength in adults of all ages through the 10th decade of life [2]. Because of increased muscle mass, research has shown that resistance training can also increase the resting metabolic rate [3]. Some studies have indicated that resistance training is as effective as aerobic endurance training in reducing major cardiovascular disease risk factors [4]. Furthermore, resistance training research has produced evidence about reversing aging factors. This means that resistance training may revert aging back to younger levels for certain genes affected by both age and exercise. Specifically, in a study by Menlov et al., favorable changes were observed in numerous genes associated with age and strength training [5].

One mechanism in which strength training may be able to reduce disease and slow the aging of cells is by preserving the length of telomeres. Telomeres are the DNA protein caps that provide stability and shield the ends of chromosomes [6]. When cells divide in mitosis, a portion of the telomeric DNA does not replicate. With the shortening of telomeres, cell senescence increases. Because somatic cells experience a finite number of cell divisions, telomere length is highly related to chronological age [7]. It reflects a person’s telomere length when born and subsequent telomere shortening. Hence, the shortening of telomeres is an index of cell aging [8]. 

In general, telomere shortening contributes to biological aging. Telomere shortening can be hastened by several factors that promote inflammation and oxidative stress. For example, obesity [9], smoking [10], poor diet [11], Type 2 diabetes [12], and low socioeconomic levels [13] are all predictive of shorter telomeres in adults. 

There is a plethora of evidence showing that telomeres are a good index of cell aging and mortality. Cawthon analyzed data from older adults over 15 years [14]. The results showed that individuals with shorter telomeres had 1.9-fold greater all-cause mortality compared to those with longer telomeres. Swedish twins who had shorter telomeres compared with their co-twin had approximately threefold higher mortality over 7 years compared with the co-twin [15]. Finally, in a study of 510 sample pairs, the results showed that shorter telomeres at baseline were predicative of greater all-cause mortality over 10 years [16]. Specifically, those who survived during the 10 years of follow-up had telomeres that were approximately 50% longer (median) than those who died. 

The present study sought to investigate the association between weekly time spent strength training and leukocyte telomere length. Regular strength training seems to be related to increased health and function of cells, but the degree to which strength training diminishes cellular aging is currently unknown. 

The aim of this investigation was to evaluate the degree to which strength training accounted for differences in telomere length in an NHANES sample of 4814 women and men representing the adult population of the US. An ancillary purpose was to determine the extent to which the association of strength training and telomere length was influenced by differences in age, sex, race/ethnicity, income, household size, smoking packyears, body mass index (BMI), and participation in physical activities other than strength training. 

## 2. Materials and Methods

### 2.1. Study Design and Sample

The National Health and Nutrition Examination Survey (NHANES) is an ongoing study conducted by the Centers for Disease Control and Prevention (CDC) that provides information about the lifestyle, health, and nutrition status of US civilians. 

A multifaceted sampling design was employed by NHANES to enable the findings to be generalized throughout the United States [17]. Specifically, NHANES employed a four-stage sampling strategy, with random selection used at all 4 stages. First, counties were selected, followed by city blocks or roads, then households were chosen, and lastly individuals were selected for participation [17].

The design of the current study was cross-sectional. NHANES data with telomere length values were only collected over a 4-year period, 1999–2002. During this period, multiple subcategories were oversampled to afford more exact estimates, including low-income individuals, Mexican Americans, non-Hispanic Black Americans, and individuals aged 60 or older. All the NHANES datasets are posted online and are available to the public for free [18]. The Ethics Review Board for the US National Center for Health Statistics (NCHS) approved the NHANES procedures (Protocol #98-12) [19]. The files posted online by NHANES contain no confidential information. Each participant provided written consent to take part in the national survey. 

During the four years that leukocyte telomere length was measured by NHANES, all participants 20 years and older were asked to give a DNA sample. In total, 7827 adults of 10,291 potential participants provided a viable blood sample that could be used to measure leukocyte telomere length. With the participants delimited to adults 20–69 years old, the sample was reduced to 6061 individuals. Women who were pregnant were not included, resulting in a sample of 5826. Participants with missing data were not part of the study, leaving 4815 adults. One individual had a telomere length that was more than 10 standard deviations above the mean and was not included, leaving a final sample of 4814 adults. 

### 2.2. Telomere Length

Telomere length data were collected by an assay using the quantitative PCR method. The assays were performed in the Blackburn laboratory at the University of California, as described elsewhere [20,21]. The process used to measure the length of telomeres has been described thoroughly by NHANES. Specifically,

“Each sample was assayed 3 times on 3 different days. The samples were assayed on duplicate wells, resulting in 6 data points. Sample plates were assayed in groups of 3 plates, and no 2 plates were grouped together more than once. Each assay plate contained 96 control wells with 8 control DNA samples. Assay runs with 8 or more invalid control wells were excluded from further analysis (<1% of runs). Control DNA values were used to normalize between-run variability. Runs with more than 4 control DNA values falling outside 2.5 standard deviations from the mean for all assay runs were excluded from further analysis (<6% of runs). For each sample, any potential outliers were identified and excluded from the calculations (<2% of samples). The mean and standard deviation of the T/S ratio were then calculated normally”.

[21]

### 2.3. Strength Training

Individuals were asked to report their level of participation in 48 different physical activities during the past 30 days, including weightlifting. Minutes spent strength training per week was treated as a categorical variable with three levels, and as a continuous variable for a different set of analyses. Adults participating in less than 10 min of strength training per week were categorized into the non-strength training group. Adults who engaged in 10–50 min of resistance training per week were categorized into the moderate resistance training group. Adults who participated in 60 min or more of strength training weekly were placed in the high strength training group.

### 2.4. Covariates 

#### 2.4.1. Race

NHANES grouped adults into five racial/ethnic groups: non-Hispanic White, non-Hispanic Black, Mexican American, other Hispanic, and other race/multiracial.

#### 2.4.2. Body Mass Index

BMI was calculated as weight in kilograms divided by height in meters squared. BMIs less than 18.5 were considered underweight. Normal BMI was between 18.5 and less than 25. The overweight category ranged from 25 to less than 30, and a BMI of 30 or more was considered obese.

#### 2.4.3. Income

NHANES reported income in USD 5000 values from USD 0 to USD 75,000. This study collapsed the income categories and reported them as less than USD 25,000, USD 25,000–45,000, USD 45,000–65,000, and USD 65,000 or more. Participants who did not report their income were included in a “missing” category. 

#### 2.4.4. Household Size

Household size was reported by integrals of one from 1 to 6, with additional household members placed into the highest category of 7+ household members. 

#### 2.4.5. Smoking Packyears

Cigarette smoking was treated as a categorical variable. Packyears were calculated by multiplying the number of cigarette packs smoked per day by the number of years the person had smoked [22].

#### 2.4.6. MET-Minutes of Physical Activity

To quantify the amount of physical activity reported other than strength training, MET-minutes were calculated on the basis of participation in any of 47 other physical activities during the past 30 days [23]. Each activity was reported to have either moderate or vigorous intensity, based on NHANES’s definitions. Depending on if the activity was reported as moderate or vigorous intensity, each mode of activity was given predetermined MET values. Participants also disclosed the number of times and average duration of each activity over the past 30 days. 

The MET values allocated by NHANES for the 48 physical activities, including strength training, are reported on the NHANES website [23]. Total MET-minutes were calculated by summing the MET-minutes for each activity (not including strength training), and a weekly MET-minute score was then tallied for each participant. 

### 2.5. Statistical Analyses

NHANES includes clusters, strata, and individual sample weights with their data so that the results can be generalized to the US adult population. In the present study, each statistical analysis used weighted data. The sample weights provided an adjustment for the unequal selection probability, non-responses, and independent population controls. 

Given the large sample size of the present investigation, the statistical power would be assumed to be excellent. However, because of the multilevel sampling process employed by NHANES, statistical power was decreased significantly. Specifically, the degrees of freedom in the denominator were calculated by subtracting the number of strata (28) from the number of clusters (57), not the number of participants. Therefore, the degrees of freedom in the study were 29 rather than several thousand. 

Weighted frequencies were generated using the SAS SurveyFreq procedure. Weighted means were calculated using the SAS SurveyMeans procedure. By employing multiple regression (SAS SurveyReg) and one-way analysis of variance (ANOVA), the extent to which mean telomere lengths differed between the frequent and moderate lifters, and the non-lifters across the strength training categories was calculated. Potential confounding variables were controlled statistically using partial correlation. Mean telomere lengths were adjusted on the basis of the differences in the covariates using the LSmeans procedure. When both minutes of strength training and telomere length were treated as continuous variables, the relationship between the two was measured by using weighted regression analysis and the SAS SurveyReg procedure. Mean differences were considered significant when *p* < 0.05. SAS software (version 9.4) was used to analyze the data.

## 3. Results

There were 4814 adults in the sample. The sample included men and women aged 20–69 years. The mean age (±SE) was 42.9 (±0.4) years. The mean telomere length was 5890 (±40) base pairs. Table 1 shows the number, percentage, and standard error of the percentage for each of the categorical variables.

### 3.1. Strength Training and Telomere Length

As shown in Table 2 (Model 1), after adjusting for the demographic covariates, there were significant differences in the mean telomere lengths across the three strength training categories (F = 6.9, *p* = 0.013). Adults who strength trained for one hour or more per week (the highest category) had significantly longer telomeres than those who did not engage in strength training. Additionally, adults who reported some strength training, but less than one hour per week, had significantly longer telomeres than the non-strength trainers. Men and women in the highest strength training category had telomeres that were 238 base pairs longer than those of non-lifters, and those in the moderate strength training category had telomeres that were 140 base pairs longer than those of non-strength trainers. 

In Table 2 (Model 2), after adjusting for all the demographic and lifestyle covariates, there were significant differences in telomere lengths across the three strength training categories (F = 6.2, *p* = 0.019). Moderate lifters and those in the highest lifting category each differed from the non-lifters in telomere length. The mean difference was 225 base pairs between the two extreme categories, and 123 base pairs between those in the moderate lifting category and those reporting no regular strength training. Adults who trained the most also had longer telomeres than the moderate group, reaching borderline significance. Again, those who trained more than an hour per week had the longest telomeres, and the non-strength trainers had the shortest telomeres. 

When time spent strength training and telomere length were both treated as continuous variables, the relationships were strong, linear, and positive. Specifically, with the demographic covariates controlled statistically, for each 10 min increase in strength training, telomeres were 7.1 base pairs longer, on average (Table 3). Similarly, after adjusting for all the covariates, for each 10 min increase in time engaged in strength training per week, the mean telomere length was 6.7 base pairs longer, on average (Table 3). 

### 3.2. Covariates and Telomere Length

Chronological age was strongly related to the length of telomeres. Specifically, as chronological age increased, telomere length decreased linearly (F = 120.8, *p* < 0.0001). For each year of chronological age, telomeres were 15.5 base pairs shorter, on average. The relationship between age and telomere length was similar for US men and women. In men, for each one-year increase in chronological age, telomeres were 15.8 base pairs shorter. In women, for each one-year increase in age, telomeres were 15.3 base pairs shorter. There was no significant difference in the length of telomeres between men and women after controlling for differences in chronological age (F = 1.8, *p* = 0.1931). 

After adjusting for differences in chronological age, the length of telomeres was associated with race (F = 4.9, *p* = 0.0037). Specifically, non-Hispanic Whites had the shortest telomeres and those reporting their race as Mexican American or multiracial had the longest telomeres. Family size was associated with telomere length (F = 3.0, *p* = 0.0199). Families reporting four to five total household members had the longest telomeres and those with smaller or larger families had shorter telomeres. Total income level was not related to telomere length (F = 1.8, *p* = 0.1630), but the body mass index (BMI) was associated with telomere length (F = 3.0, *p* = 0.0359). Specifically, normal weight adults had longer telomeres than those with obesity. Total MET-minutes summed across 47 different physical activities (not including strength training) were not predictive of telomere length (F = 0.1, *p* = 0.7927). Lastly, the variable of smoking packyears was highly related to telomere length (F = 19.8, *p* < 0.0001). The more packyears accumulated by individuals, the shorter their telomeres were. 

## 4. Discussion

The overall purpose of the study was to determine the relationship between strength training, measured by time spent strength training per week, and telomere length, a measure of biological aging. The nationally generalizable results were generated from 4814 randomly selected US men and women aged 20–69 years. 

Previous studies of strength training have focused on factors such as loading resistance [24], muscle strength, and hypertrophy [25,26,27], and strength training as a treatment for long-term health [28,29]. To date, the relationship between time spent strength training and telomere length has not been evaluated in a large, population-based investigation. 

In a study with a small sample, telomere lengths were evaluated in seven power lifters and compared with a group of seven healthy non-lifters to determine if the practice of intense resistance training is related to telomere length [30]. The study found that telomere restriction fragments were higher in powerlifters than in the control group, and there was no abnormal telomere shortening in the powerlifters [30]. However, the powerlifting investigation focused on a unique group of intense lifters who differed considerably from the general public, given that the powerlifters’ training spanned 8 ± 3 years. 

Given the natural shortening that occurs in telomeres with age, the present study findings can be interpreted in greater detail. With both variables treated as continuous, as shown in Table 3, as time spent strength training increased, the telomeres were significantly longer, signifying a linear association. After adjusting for differences across all the covariates, the findings showed that for each 10 min spent strength training per week, telomeres were 6.7 base pairs longer, on average. Therefore, 90 min per week of strength training was predictive of telomeres that were 60.3 base pairs longer, on average (9 × 6.7= 60.3). Because each year of chronological age was associated with telomeres that were 15.47 base pairs shorter in this national sample, 90 min per week of strength training was associated with 3.9 years less biological aging, on average (60.3/15.47 = 3.9). This interpretation suggests that an hour of strength training three times per week (180 total minutes) was associated with 7.8 years less biological aging. 

Explaining the outcome that regular strength training predicts less cellular aging begins with evaluating the mechanisms associated with telomeres and biological function. In general, lifestyle factors such as obesity, exercise, and diet have been linked to biological aging and telomere health [31]. Moreover, regular strength training results in many health benefits that might account for its relationship with longer telomeres and reduced biological aging. In a comprehensive article by Westcott [1], the benefits of resistance training related to biological aging and overall health include reversing muscle loss, increasing resting metabolic rate, reducing body fat, and improving cardiovascular health. Strength training has also been found to decrease oxidative stress [32] and therefore may serve as a therapeutic strategy to reduce the pace of aging. 

Biological aging is accompanied by the loss of muscle mass and a decrease in the resting metabolic rate of skeletal muscle. Sarcopenia involves the loss of muscle tissue accompanying the aging process that is responsible for decreases in strength and functional deficiency in the aging adult. Sarcopenia is associated with adverse outcomes, such as falls, functional decline, frailty, and mortality [33,34,35], as well as increasing health problems, such as sarcopenic obesity and diabetes [36]. 

Strength training has been shown to reverse the decline in skeletal muscle. Several studies have concluded that strength training programs lead to increases in strength as well as increases in muscle mass throughout all ages [2,37,38,39]. One large-scale study found that there was a mean lean body increase of 1.4 kg after a consistent 10-week strength training program [39]. Strength training is a practical option to retain muscle mass and prevent the strength loss that coincides with biological aging.

Sarcopenia affects the resting metabolic rate. With the significant increases in muscle mass that accompany regular strength training, the mean energy intake required for body weight and tissue maintenance can increase by 15% [2]. Some suggest that a 1.0 kg increase in trained muscle tissue may raise the resting metabolic rate by about 20 kilocalories per day [4]. With more energy at rest required for tissue maintenance, resistance training tends to stimulate muscle protein turnover [40,41] and increase arterialized plasma norepinephrine levels [42]. Some evidence has indicated that the maintenance of a larger muscle mass might decrease metabolic risk factors such as obesity, dyslipidemia, and Type 2 diabetes mellitus [43,44]. In a study by Heden [45], beginning participants who performed either moderate-volume resistance training or low resistance training averaged a 5% increase in resting metabolic rate for a few days after the resistance sessions [45].

Aging factors are amplified in the presence of excessive body fat. Overweight and obesity are associated with risk factors such as hypertension and hyperlipidemia that promote the development of Type 2 diabetes and cardiovascular disease [46,47]. Regular resistance training appears to alter body composition in men and women. Multiple resistance training studies have resulted in lean muscle gains with significant fat weight loss [2,38,48].

Intra-abdominal fat resides beneath the abdominal wall and tends to be significantly reduced with strength training. In several investigations, both older women and older men were found to have decreases in visceral fat from weightlifting [49,50,51,52]. Increased metabolic rate, improved insulin sensitivity, and enhanced sympathetic activity are proposed mechanisms by which resistance training decreases visceral fat stores [53]. In one study, the treatment group reduced body fat in comparison with the control group by performing strength training twice a week for two years [54].

Longer telomeres may result from improved cardiovascular health due to regular strength training [55]. Several studies have evaluated resting blood pressure within a strength training program and have produced evidence of lowered resting systolic and diastolic blood pressure [39,56,57]. Research has shown that the typical drop in resting systolic and diastolic blood pressure readings with strength training is about 6.0 mm Hg systolic and 4.7 mm Hg diastolic [58]. Additionally, in other research, strength training has been shown to be an effective means to improve low density lipoprotein cholesterol, high density lipoprotein cholesterol, and triglyceride profiles [59,60,61,62]. 

In summary, evidence demonstrates the effectiveness of resistance training in important areas of physical health. Biological aging is accelerated through chronic diseases such as obesity, diabetes, hypertension, hypercholesterolemia, and cardiovascular disease. As strength training mitigates some of the damage caused by such chronic diseases, reversing muscle loss, raising resting metabolic rate, promoting fat loss, and improving cardiovascular health, it is logical that strength training may limit disease and slow the aging of cells. In short, by reducing the effects of chronic disease and metabolic risk factors, resistance training appears to slow the biological aging process and reduce cell senescence, which is evidenced by longer telomeres. 

There were multiple limitations within the present study. First, NHANES employed a cross-sectional design, eliminating the possibility of finding a cause-and-effect relationship. Time spent strength training was strongly related to lower levels of biological aging, but it cannot be said that regular strength training caused the length of telomeres to be longer. Second, time spent strength training was self-reported, so there is a possible influence of misclassification of the amount of strength training. In general, self-reporting tends to increase measurement error, so if an objective measure of strength training were employed, the relationship between strength training and telomere length would likely have been stronger. Also, the strength training measure focused on strength training during the past 30 days, so long-term strength training information could not be evaluated. Additionally, participants within the high and moderate levels of strength training could have other lifestyle behaviors unique to their subgroups that influenced the favorable findings. All possible covariates could not be measured and controlled statistically.

The present study also had multiple strengths. First, a reputable lab with excellent established methods was used for the measurement of telomere length, independent of the present study. Second, a variety of demographic and lifestyle variables were controlled statistically, which minimized their effect on the relationship between strength training and telomere length. Third, telomere length was highly related to chronological age, which it should be if measured accurately. Finally, the sample was large, randomly selected, multi-racial, and representative of the US population 20–69 years of age using a multistage probability sampling design. 

## 5. Conclusions

The length of telomeres is a good index of cellular aging. In the present study, mean telomere lengths differed significantly between US adults reporting no strength training and those reporting moderate or high amounts of strength training. Participants reporting the most time spent strength training had significantly longer telomeres than those in the no strength training category. The difference in cellular aging was substantial. After adjusting for all the potential cofounders, for each 10 min increase in time engaged in strength training per week, the mean telomere length was about 6.7 base pairs longer, on average, compared with non-lifters. This was equivalent to almost 4 years less biological aging for adults participating in about 90 min of strength training per week. Based on a random sample of almost 5000 women and men, representative of the US adult population, the findings indicate that regular strength training and longer telomeres are meaningfully and significantly related.

## Figures and Tables

**Table 1:** Characteristics of the sample based on the categorical variables (N = 4814).

Categorical Variable	N	%	SE
**Sex**			
Women	2475	51.4	0.52
Men	2339	48.6	0.52
**Race/ethnicity**			
Mexican American	382	7.9	0.86
Other Hispanic	331	6.9	1.56
Non-Hispanic White	3417	71.0	2.04
Non-Hispanic Black	515	10.7	1.34
Other race/multiracial	169	3.5	0.56
**Body mass index**			
Underweight	78	1.6	0.18
Normal weight	1498	31.1	0.77
Overweight	1595	33.1	1.16
Obese	1537	32.0	1.07
Missing	106	2.2	0.32
**Strength training**			
None	4480	93.1	0.73
10–50 min per week	149	3.1	0.45
≥60 min per week	185	3.8	0.38
**Household size**			
1	450	9.4	0.47
2	1436	29.8	1.33
3	1002	20.8	1.25
4	988	20.5	1.25
5	505	10.5	0.76
6	183	3.8	0.69
7+	250	5.2	0.69
**Income**			
USD < 25,000	1121	23.3	1.17
USD 25,000–44,999	914	19.0	1.07
USD 45,000–64,999	805	16.7	0.86
USD 65,000 or more	1516	31.5	1.84
Missing	458	9.5	1.11

Note: SE refers to the standard error of the percentage. N means the number of subjects, and the % column indicates the percentage of participants in each category. Both the numbers and percentages reflect the values after person-level sample weights were applied. Weighted values were used because they can be generalized to the US population.

**Table 2:** Differences in mean telomere length (base pairs) by level of strength training in US adults, after adjusting for the covariates (N = 4814).

	Strength Training Time				
	None	10–50 min/Week	≥60 min/Week		
Model:	Telomere Mean ± SE	Telomere Mean ± SE	Telomere Mean ± SE	F	*p*
Model 1	5876 ^a^ ± 37	6016 ^b^ ± 72	6114 ^b^ ± 121	6.9	0.013
Model 2	5894 ^a^ ± 38	6017 ^b^ ± 79	6119 ^c^ ± 122	6.2	0.019

Note: Means on the same row with the same superscript letters, a, b, or c, were not significantly different. The strength training categories were defined as follows: None: adults who participated in less than 10 min per week of strength training (N = 4480, 93.1%); 10–50 min/week: adults who participated in 10–50 min per week of strength training (N = 149, 3.1%); ≥60 min/week: adults who participated in 60 min or more per week of strength training (N = 185, 3.8%). Model 1 controlled for differences in the demographic variables of age, sex, race, income, and household size. Model 2 evaluated the mean differences in telomere lengths after controlling for the demographic factors and also the lifestyle covariates (smoking packyears, BMI, and MET-minutes of physical activity other than strength training). In Model 2, the difference between the moderate and high strength training groups was borderline significant (*p* = 0.0897).

**Table 3:** The association between time spent strength training and telomere length in 4814 US adults.

	Telomere Length				
Predictor	Model	Regression Coefficient (Slope)	SE	F	*p*
Strength training (time)	1	7.1	1.9	14.7	0.0006
	2	6.7	1.8	14.7	0.0006

Note: Model 1 included adjustment for differences in the demographic covariates of age, sex, race, household size, and income. Model 2 controlled for differences in the demographic covariates and smoking packyears, BMI, and MET-minutes spent in physical activities other than strength training. The interpretation of the Model 1 results would be that for each 10 min US adults participated in strength training per week, their telomeres were 7.1 base pairs longer, on average.

## Data Availability

The original data presented in the study are openly available in the repository “NHANES Questionnaires, Datasets, and Related Documentation” at https://wwwn.cdc.gov/nchs/nhanes/Default.aspx (accessed on 28 October 2024).
